# Dietary non-fermentable fiber prevents autoimmune neurological disease by changing gut metabolic and immune status

**DOI:** 10.1038/s41598-018-28839-3

**Published:** 2018-07-11

**Authors:** Kerstin Berer, Inés Martínez, Alesia Walker, Birgit Kunkel, Philippe Schmitt-Kopplin, Jens Walter, Gurumoorthy Krishnamoorthy

**Affiliations:** 1 Hertie Senior Professor Group, Max Planck Institute of Neurobiology, Martinsried, Germany; 2 Department of Agricultural, Food & Nutritional Science, University of Alberta, Edmonton, Canada; 3 Research Unit Analytical BioGeoChemistry, Helmholtz Zentrum München, Neuherberg, Germany; 4 Chair of Analytical Food Chemistry, Technische Universität München, Freising-Weihenstephan, Germany; 5 ZIEL – Institute for Food & Health, Technische Universität München, Freising, Germany; 6 Department of Biological Sciences, University of Alberta, Edmonton, Canada; 7 Research group Neuroinflammation and mucosal Immunology, Max Planck Institute of Biochemistry, Martinsried, Germany

## Abstract

The autoimmune neurological disease, Multiple Sclerosis (MS), have increased at alarming rates in the Western society over the last few decades. While there are numerous efforts to develop novel treatment approaches, there is an unmet need to identify preventive strategies. We explored whether central nervous system (CNS) autoimmunity can be prevented through dietary manipulation using a spontaneous autoimmune encephalomyelitis mouse model. We report that the nutritional supplementation of non-fermentable fiber, common components of a vegetarian diet, in early adult life, prevents autoimmune disease. Dietary non-fermentable fiber alters the composition of the gut microbiota and metabolic profile with an increase in the abundance of long-chain fatty acids. Immune assays revealed that cecal extracts and a long chain fatty acid but not cecal lysates promoted autoimmune suppressive T_H_2 immune responses, demonstrating that non-fermentable fiber-induced metabolic changes account for the beneficial effects. Overall, these findings identify a non-invasive dietary strategy to prevent CNS autoimmunity and warrants a focus on nutritional approaches in human MS.

## Introduction

The dietary habits of humans have changed dramatically in the last few decades, notably in industrialized western countries and in urban societies. Parallel to these changes, there has been a considerable increase in autoimmune diseases that include multiple sclerosis (MS)^1,2^. As opposed to people living in societies with a traditional lifestyle, people in industrialized countries consume diet with low dietary fiber and high fat content^3^. It is believed that “Western diet” alters gut microbiome composition and functions, thereby affecting autoimmune disease pathogenesis^3,4^. Thus, identifying disease relevant dietary elements will guide us devising strategies not only to treat but also prevent autoimmune diseases.

Dietary fibers, comprise of complex carbohydrates that can be either soluble (e.g.: pectin) or insoluble (e.g. cellulose)^5^, have a wide range of physiological effects, and are considered crucial for human health. Indeed, several recent reports showed that the end-products of the fiber fermentation, short chain fatty acids (SCFAs), shape the immunological microenvironment of the gut and have protective functions in several autoimmune and allergic diseases^6–10^. However, most insoluble fibers, such as cellulose, are an abundant component of most plant tissues including vegetables and fruits, and are poorly digested by the gut microbiota of non-ruminant mammals^11–13^. Despite the notion that non-fermentable fiber components can modulate microbiome composition^14,15^, their role in autoimmune disease pathogenesis is unclear.

In this report, we investigated the effects of non-fermentable dietary fiber consumption on the pathogenesis of central nervous system (CNS)-specific autoimmune disease, using a genetically engineered spontaneous experimental autoimmune encephalomyelitis (EAE) mouse model. We demonstrate, for the first time, that non-fermentable dietary fiber consumption protects mice from developing spontaneous CNS-directed autoimmunity. Interestingly, this protective effect can be reversed by simply switching diet to a low fiber diet during early adult life. The disease protection went along with robust changes in microbiota composition, metabolic profile and induction of T_H_2 immune responses within and outside the intestine. Together, these finding establish that dietary non-fermentable fiber as a modulator of gut microbial profile and offer a simple way to prevent CNS autoimmunity that warrants nutritional studies in human MS.

## Results

### Dietary non-fermentable fiber suppresses spontaneous CNS autoimmunity

**Figure 1:**
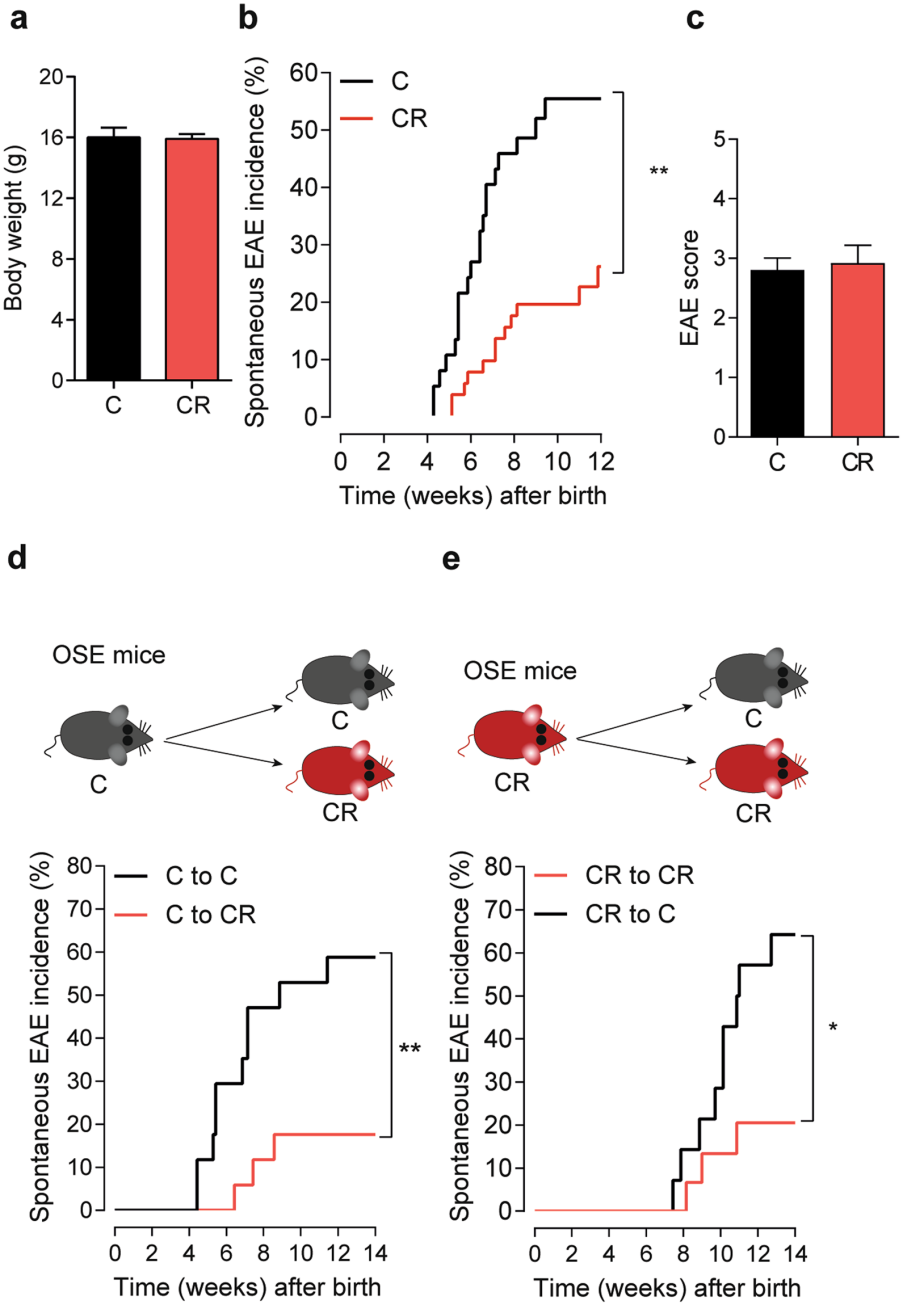
Diet rich in cellulose suppresses spontaneous EAE in OSE mice. (**a**) Body weight is not altered by high dietary cellulose content. Bar graph displays the body weight of 5 weeks old OSE mice fed control (C; *n* = 18) or cellulose rich (CR) diet (CR; *n* = 55). (**b**) Incidence of spontaneous EAE in OSE mice fed control (C; *n* = 37) or CR diet (CR; *n* = 51). ***P < 0.001 (Gehan-Breslow-Wilcoxon test). (**c**) Mean EAE scores of OSE mice that showed neurological symptoms. Control (C; *n* = 15) or CR diet (CR; *n* = 6). (**d**–**e**) Incidence of spontaneous EAE after diet switch in early life. (**d**) OSE mice raised on control diet were either weaned on to control (C to C; *n* = 17) or on to CR diet (C to CR; *n* = 17) at 4 weeks of age. **P < 0.01 (Gehan-Breslow-Wilcoxon test). (**e**) OSE mice raised on CR diet were either weaned on to CR (CR to CR; *n* = 15) or on to control diet (CR to C; *n* = 14) at 4 weeks of age. **P < 0.05 (Gehan-Breslow-Wilcoxon test).

We sought to address the influence of non-fermentable dietary fiber on CNS-specific autoimmune disease using a spontaneous experimental autoimmune encephalomyelitis (EAE) mouse model, opticospinal encephalomyelitis (OSE) mice^16^. We chose a spontaneous EAE mouse model over classic active EAE models to avoid exogenous influence of adjuvants on the microbiome and skewing of the immune responses^17^. Mice were raised on a standard rodent diet or a crude fiber-rich diet, high in cellulose (26%) (Cellulose rich, CR). No differences in body weights (Fig. 1a), litter size, or gross behavior were noted between the groups of mice.

We followed OSE mice raised on control or CR diet for the development of spontaneous neurological symptoms. While the spontaneous EAE (sEAE) incidence was approximately 55% in control diet-fed mice, OSE mice on CR diet showed a significantly lower EAE incidence (23%) with a delayed onset of neurological symptoms (Fig. 1b). However, we did not find any differences in disease severity (Fig. 1c) or expression of inflammatory markers in the spinal cord (Supplementary Fig. 1a**)** among mice that have developed EAE. To determine whether CR diet-induced disease protection is reversible by simply switching the diet during early adult life, we performed diet switch experiments. Litters of OSE mice, raised on control diet, were either weaned between 3–4 weeks after birth onto control diet or onto CR diet. Analogous diet switch experiments were performed from litters of CR diet-fed OSE mice. Remarkably, OSE mice, which were raised on control diet but weaned onto CR diet, remained protected from the development of sEAE (Fig. 1d). Their littermates, however, reared on control diet, showed clinical neurological symptoms and a 60% disease incidence (Fig. 1d). The opposite diet-switch experiment, OSE litters raised on CR diet but weaned onto control diet, developed sEAE with comparable disease incidence and severity to mice reared on control diet. However, mice raised and weaned onto CR diet remained protected from the development of clinical neurological symptoms (Fig. 1e).

### Altered immune responses after dietary cellulose supplementation

**Figure 2:**
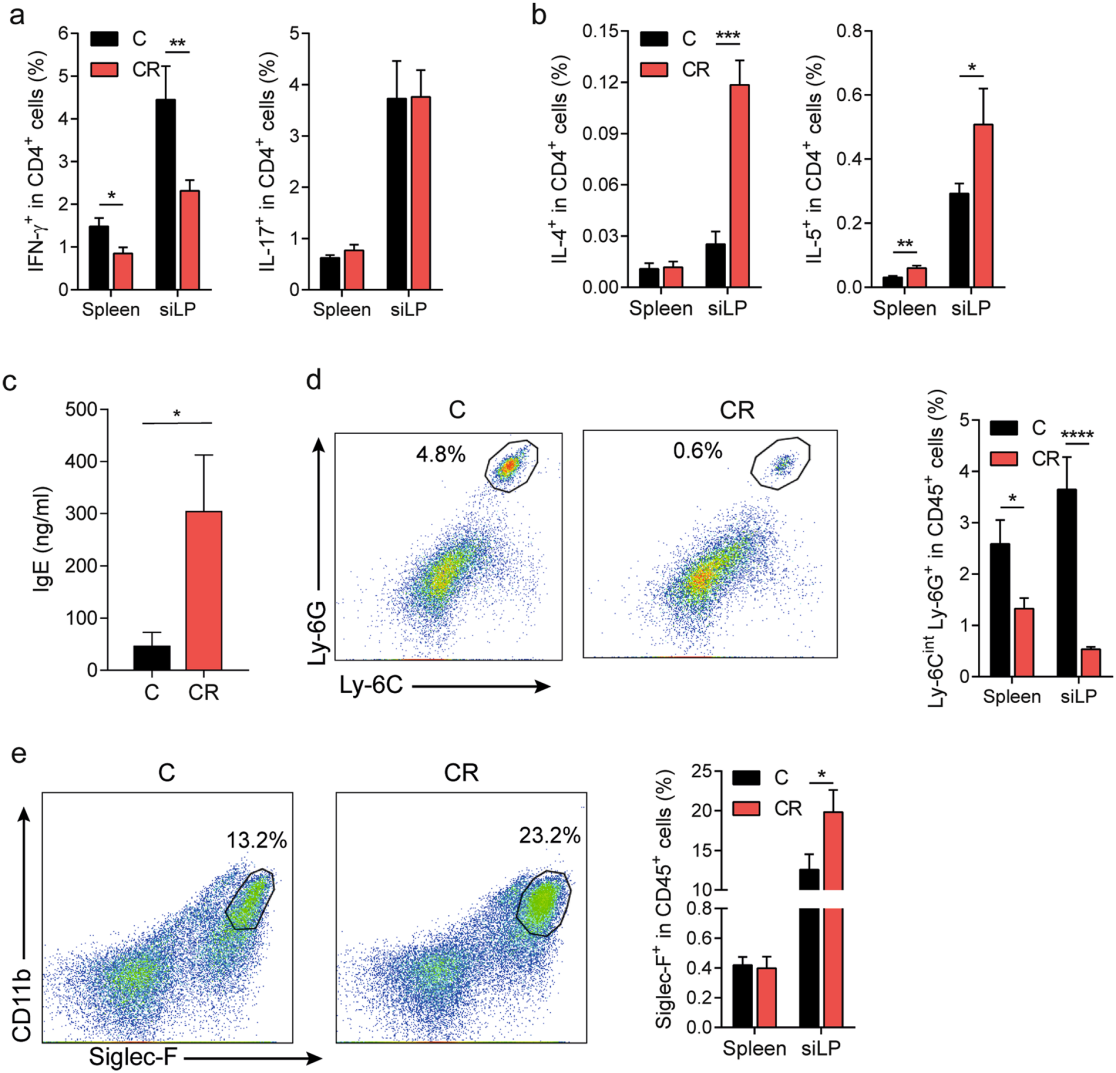
High dietary cellulose content shifts the balance from a pro-inflammatory T_H_1 towards an anti-inflammatory T_H_2 cell response. (**a**) Impaired T_H_1 immune response in OSE mice on CR diet. Frequencies of IFN-γ- and IL-17-producing T cells from the indicated organs of mice fed control (C) or CR diet are displayed. siLP, small intestinal lamina propria. *n* = 9–12 mice per group. Data were pooled from 3 independent experiments. **P < 0.01; *P < 0.05 (Mann-Whitney U test). (**b**) Enhanced T_H_2 cell responses in CR diet-fed OSE mice. Shown are the frequencies of IL-4- and IL-5-producing T cells from the indicated organs of mice fed control (C) or CR diet. *n* = 8–9 mice per group. Data were pooled from 2 independent experiments. ***P < 0.001; **P < 0.01; *P < 0.05 (Mann-Whitney U test). (**c**) Serum IgE levels measured by ELISA. Bars display mean ± s.e.m. *n* = 10–11 mice per group. Data were pooled from 2 independent experiments. *P < 0.05 (Mann-Whitney U test). (**d**) Lack of neutrophil recruitment in OSE mice on CR diet. Representative plots of cells from small intestinal lamina propria (siLP) of OSE mice raised on control (C) or CR diet. Ly-6C^int^Ly-6G^+^ populations of CD45^+^ cells are gated and their percentages are shown. Bar graph depicts frequencies of neutrophils (CD45^+^Ly-6C^int^Ly-6G^+^) from the indicated organs of mice fed C or CR diet. *n* = 7–9 mice per group. Data were pooled from 2 independent experiments. ****P < 0.0001; *P < 0.05 (Mann-Whitney U test). (**e**) Increased frequencies of eosinophils in CR diet-fed OSE mice. Representative plots of cells from siLP of OSE mice raised on control (C) or CR diet. Siglec-F^+^ populations of CD45^+^ cells are gated and their percentages are shown. Bar graph displays frequencies of eosinophils (CD45^+^Siglec-F^+^) from the indicated organs of mice fed C or CR diet. *n* = 8–9 mice per group. Data were pooled from 2 independent experiments. *P < 0.05 (Mann-Whitney U test).

To identify the molecular mechanisms resulting in suppression of CNS-specific autoimmune disease, we sorted CD4^+^ T cells from the intestinal lamina propria of control and CR diet-fed mice and determined their cytokine expression profile by qPCR screens. The analysis demonstrated that the pathogenic T cell response was diminished in mice on CR diet, since the expression levels of the signature T_H_1 cell-cytokine IFN-γ as well as its transcription factor T-bet were decreased in intestinal CD4^+^ T cells from CR compared to control diet-fed animals (Supplementary Fig. 1b). Moreover, T cells from mice fed a CR diet expressed higher transcripts levels of the T_H_2 cell-associated cytokines IL-4 and IL-5 compared to T cells from control diet-fed mice (Supplementary Fig. 1b). We verified the qPCR screening results by flow cytometric analysis of T cells isolated from the intestine as well as the spleen. Consistent with our qPCR findings, mice raised on CR diet had 50% less IFN-γ-producing T_H_1 cells in the small intestinal lamina propria (siLP) than mice on control diet (Fig. 2a). Interestingly, the T_H_1 cell response was not only affected in the gut of CR diet-fed mice, but also dampened in the periphery, as analysis of CD4^+^ T cells from mice on CR diet showed a decrease in IFN-γ^+^ T_H_1 cells in the spleen (Fig. 2a). In contrast, pro-inflammatory T_H_17 cell response was not altered by the increased dietary cellulose content with control and CR diet-fed mice harboring similar frequencies of IL-17-producing T_H_17 cells in the spleen as well as the siLP (Fig. 2a). In addition, we also observed in changes in the T_H_2 immune responses. Specifically, CR diet-fed mice had increased frequencies of IL-4- and IL-5-producing CD4^+^ T cells in the small intestine and to a lesser extent in the spleen compared to mice fed a control diet (Fig. 2b). Furthermore, the elevated T_H_2 immune response was also reflected in serum IgE levels of CR diet fed mice (Fig. 2c).

In contrast to most soluble dietary fibers, cellulose remains to a large extent unfermented by the gut microbiota, resulting in negligible amounts of SCFAs being produced, which are known to induce regulatory T (T_reg_) cells^8,9^. Consistent with this, the small intestine of CR diet-fed mice harbored less T_reg_ cells compared to mice on control diet (Supplementary Fig. 1c). Since T_H_1 and T_H_2 cytokines differentially recruit innate cells, such as neutrophils or eosinophils, we analyzed the frequencies of these cell types in control and CR diet-fed mice. In line with the reduced levels of T_H_1 cell-associated cytokines as well as increased amounts of cytokines of the T_H_2 type response, we noted striking differences in the frequencies of neutrophils and eosinophils comparing mice fed a control or a CR diet. Animals reared on CR diet had approximately 2-fold less neutrophils in the spleen and 7-fold lower neutrophils in the small intestine than mice raised on control diet (Fig. 2d). On the other hand, frequencies of eosinophils were increased in mice on CR diet compared to control diet-fed animals (Fig. 2e). Of note, previous findings in MS and its animal model EAE implicate pro-inflammatory T_H_1 cells with disease induction, while T_H_2 cells have been associated with the amelioration of the disease^18–21^. Thus, we conclude that CR diet suppresses CNS-specific autoimmunity by altering T_H_1 and T_H_2 immune responses.

### Gut microbial and metabolic profiles altered by cellulose supplementation

**Figure 3:**
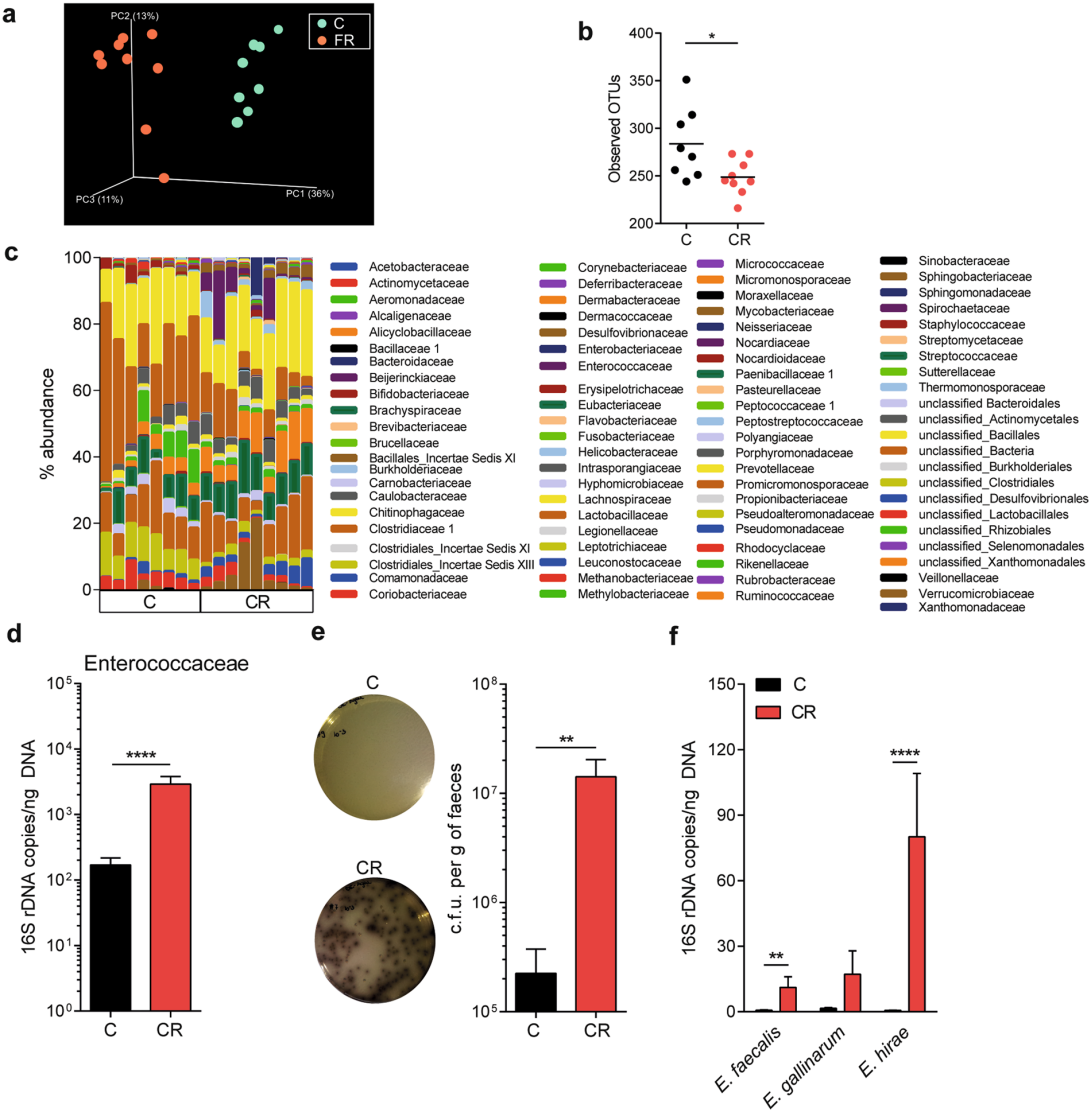
Dietary cellulose alters the composition of the intestinal microbiota. (**a–c**) 16S rRNA sequencing analysis of the cecal microbiota of OSE mice fed control or CR diet. *n* = 8–9 mice per group. (**a**) Analysis of the variance between microbial communities from the ceca of OSE mice on control (C; *n* = 8) or on CR diet (CR; *n* = 9) assessed by the average relative abundance using principal component (PC) analysis. (**b**) Phylogenetic (alpha) diversity of cecal microbiota. *P < 0.05 (Mann-Whitney U test). (**c**) Relative abundance of bacterial genera in cecal samples of mice that were fed a C or a CR diet. (**d**) Quantitative real-time PCR analysis of Enterococcaceae in the feces of OSE mice fed C or CR diet. *n* = 15–16 mice per group. ****P < 0.0001 (Mann-Whitney U test). (**e**) Quantification of Enterococci in fecal pellets by culture on Bile Esculin agar in CR diet-fed OSE mice. Bar graph depicts the CFU of Enterococci from fecal pellets of mice fed control or CR diet. *n* = 7–8 mice per group. Data were pooled from 2 independent experiments. *P < 0.05 (Mann-Whitney U test). (**f**) Outgrowth of *E*. *hirae* accounted for the increase in the *Enterococcus* genus in CR diet-fed OSE mice. Levels of *Enterococcus faecalis*, *Enterococcus gallinarum* and *Enterococcus hirae* in feces of mice on C or CR diet were determined by quantitative real-time PCR of 16s rRNA coding DNA. *n* = 15–16 mice per group. Data were pooled from 2 independent experiments. ****P < 0.0001; **P < 0.01 (Mann-Whitney U test).

The cellulose rich diet might affect CNS autoimmunity by altering the intestinal microbiota, which can modulate the immune system either directly or indirectly through metabolites. To dissect these distinct possibilities, we characterized the cecal microbiota of mice raised on either control or CR diet by 16S rRNA tag sequencing analysis. Gut bacterial communities between dietary regimens were compared in terms of diversity (alpha and beta) and composition. Beta-diversity analysis revealed distinct clustering of cecal samples according to the diet (Fig. 3a), suggesting that cellulose rich diet significantly altered the cecal overall bacterial community composition. Alpha-diversity analysis revealed that OTU richness was significantly reduced in the ceca of CR diet-fed mice (Fig. 3b). The analysis of the abundance profiles of bacterial populations showed various alterations in the microbiota of CR compared to control diet-fed mice (Fig. 3c and Supplementary Table 1). At the family level, for instance, we found an increased representation of Ruminococcaceae, Helicobacteraceae and Enterococcaceae, while the abundance of Sutterellaceae, Lactobacillaceae and Coriobacteriaceae were reduced in CR diet-fed mice (Fig. 3c and Supplementary Fig. 2a). At lower taxonomic levels, the abundance of the genera *Helicobacter*, *Enterococcus*, *Desulfovibrio*, *Parabacteroides*, *Pseudoflavonifractor* and *Oscillibacter* were increased, while *Lactobacillus*, *Parasutterella*, *Coprobacillus* and *TM7 genera Incertae Sedis* were significantly reduced in OSE mice reared on CR diet compared to control diet-fed animals (Supplementary Fig. 2b). We confirmed the increased abundance of the bacterial family Enterococcaceae by quantitative PCR using bacterial DNA isolated from fecal pellets of a different cohort of mice on control or CR diet (Fig. 3d). This increase was also confirmed by quantitative culture on Bile Esculin agar, which allows the selective growth of *Enterococcus* species (Fig. 3e). Interestingly, *Enterococcus hirae* was the dominant species present in CR diet-fed mice (Fig. 3f). This increase in Enterococci was clearly dependent on the dietary cellulose intake, since *Enterococcus* species were present in lower numbers in the intestines of control diet-fed mice with the few Enterococci detected being of the species *Enterococcus faecalis* (Fig. 3d–f).

**Figure 4:**
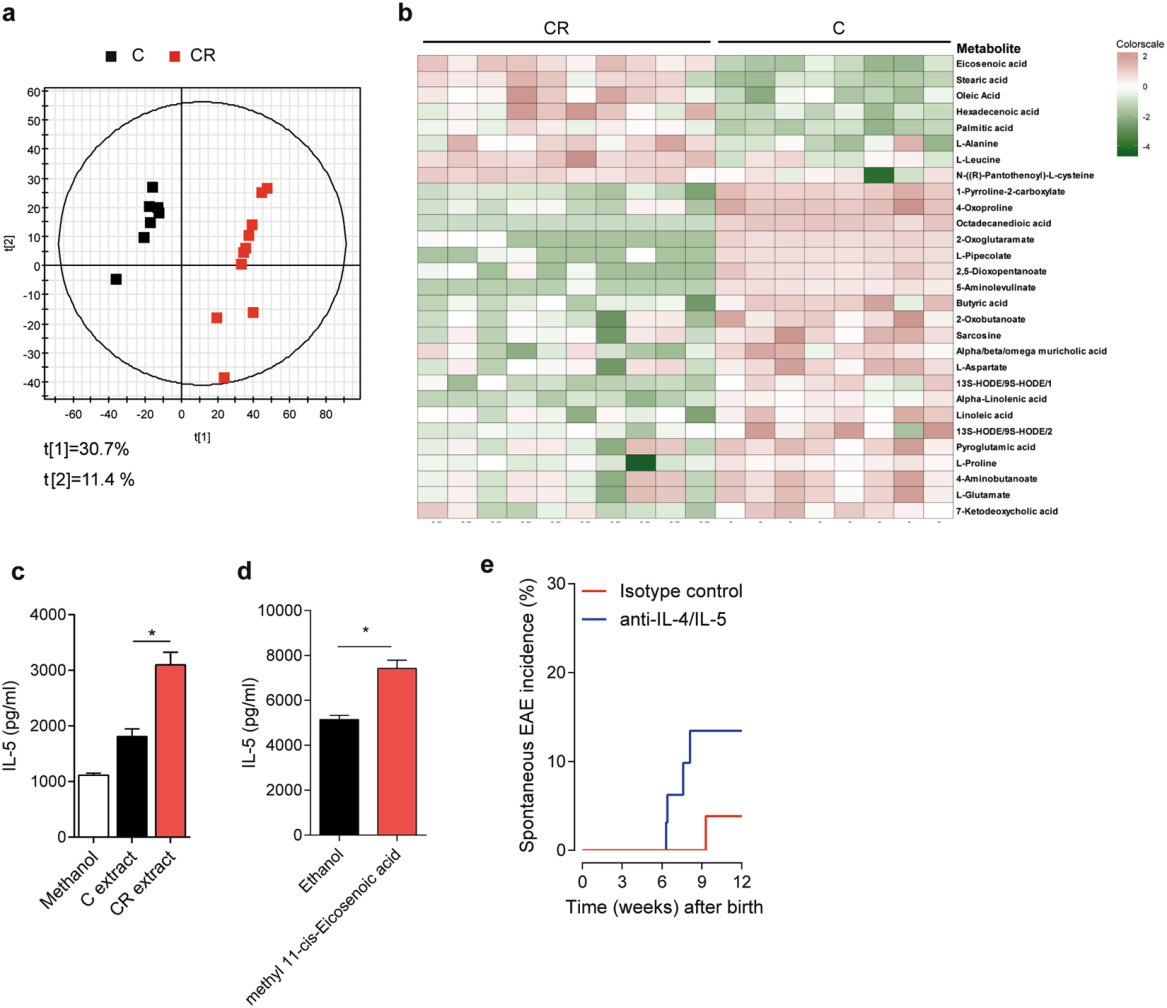
Long chain fatty acids promote type 2 immune responses. (**a**) Cecal metabolites of control (C; *n* = 8) or of CR (CR, *n* = 10) diet-fed mice were analyzed by multimodal RP-LC-MS in negative ESI mode. Cecal samples of mice reared on CR diet are clearly distinguishable from samples of control diet-fed animals in a principal component analysis. (**b**) Heat map depicts cecal metabolites differentially enriched in control (C; *n* = 8) or CR diet-fed mice (*n* = 10). (**c**) Cecal metabolites of mice fed a CR diet potentiate the T_H_2 immune response. Naïve CD4^+^ T cells were stimulated under T_H_2 cell polarizing conditions in the presence of 1% v/v methanol, cecal extracts of control (C extract) or of CR (CR extract) diet-fed mice. Levels of IL-5 in the supernatants were measured by ELISA. Bars display mean ± s.e.m. *n* = 3 independent experiments. ****P < 0.0001; *P < 0.05 (Mann-Whitney U test). (**d**) Long- chain fatty acids enhance T_H_2 immune response. Naïve CD4^+^ T cells were stimulated under T_H_2 cell polarizing conditions in the presence of ethanol or 25 µM methyl cis-11 eicosenoic acid. Levels of IL-5 in the supernatants were measured by ELISA. Bars display mean ± s.e.m. *n* = 3 independent experiments. *P < 0.05 (Unpaired t test). (**e**) Neutralization of T_H_2 cytokines, IL-4 and IL-5 partially reverses disease protection in CR-diet fed mice (Isotype control; n = 29, anti-IL-4/IL-5; n = 32). Mice were injected once weekly with 250 µg each of anti-IL-4 and & anti-IL-5 antibodies or 500 µg of isotype control antibodies. Data were pooled from 4 experiments.

Besides exerting a direct effect on the host’s immune system, gut microbes can influence immune responses indirectly via the production of metabolites. Since the increase in dietary cellulose resulted in profound changes in intestinal microbial composition, we performed non-targeted mass spectrometry (MS)-based metabolome analysis^22^ on cecal samples of OSE mice raised on control or CR diet. Principal component analysis clearly separated the dietary groups (Fig. 4a). Using KEGG pathways enrichment analysis, we determined that while valine, leucine and isoleucine biosynthesis were enhanced in CR diet-fed mice, linoleic acid metabolism was enriched in mice fed a control diet (Supplementary Fig. 3). Among the metabolites responsible for the separation, the SCFA butyric acid was significantly decreased in CR diet-fed mice (Fig. 4b). On the other hand, long-chain fatty acids, like eicosenoic acid, stearic acid, oleic acid, hexadecenoic acid and palmitic acid were increased in CR diet-fed mice (Fig. 4b). Although these fatty acids were present in in both control and CR diet, the increase of these fatty acids in CR diet fed mice might be due to the ability of cellulose to bind bile acids^23^. Bile acids facilitates the absorption of fatty acids by forming mixed micelles^24^ and lower bile acid concentrations can affect the rate of absorption of fatty acids in the intestine. Consistent with this notion, we found a significant decrease of muricholic acid, a group of bile acids, in the ceca of CR died fed mice (Fig. 4b).

### Long chain fatty acids promote T_H_2 immune responses

The increased T_H_2 cell responses seen in CR diet-fed mice might have been induced either by the altered gut microbiota, the changed metabolic profile or both. To discriminate these possibilities, we differentiated naïve T cells to T_H_2 cells *in vitro* and tested the potential of either cecal lysates (direct effect of intestinal bacteria) or cecal extracts (indirect effect via metabolites) prepared from control or CR diet-fed mice to enhance the T_H_2 cell-response. Interestingly, neither cecal bacterial lysates from mice fed a control diet nor from animals on CR diet significantly affected the T_H_2 immune response (Supplementary Fig. 4). In contrast, cecal metabolites from mice raised on CR diet clearly stimulated the T_H_2 immune response with increased production of IL-5 compared to cecal extracts from control diet-fed animals (Fig. 4c). To delineate the enhanced T_H_2 response to the changed metabolite profile in mice fed a CR diet, we tested the long chain fatty acid eicosenoic acid (methyl 11-cis eicosenoic acid) in our T_H_2 cell differentiation assays. We found that eicosenoic acid potentiated the T_H_2 cell response leading to enhanced production of IL-5 (Fig. 4d). Finally, to test if the increased T_H_2 responses contribute to disease suppression, we performed cytokine neutralization experiments. We injected blocking antibodies against T_H_2 cytokines, IL-4 and IL-5 into mice fed a CR diet. Neutralization of T_H_2 cytokines *in vivo* showed a trend towards reversal of the disease protection mediated by CR diet (Fig. 4e). Given the fact the T_H_2 cytokine neutralization did not fully restore spontaneous EAE to a level comparable to control diet, additional effects of the microbiome on T cells or other immune cells including antigen presenting cells that may have additive disease protective effects cannot be ruled out.

## Discussion

Although several lifestyle and environmental factors likely contribute to the unprecedented rise in both the incidence and prevalence of autoimmune diseases like MS in the last few decades, efforts to translate this information into therapeutic or preventive purposes has been minimal^1,25,26^. Among several lifestyle factors, diet is a potential contributor to the MS pathogenesis, and the microbiome has been suggested as a mechanism by which diet would be implicated in MS^26–28^. In addition, recent studies have shown that gut microbiome is altered in MS patients and transplantation of MS microbiota induce disease in mouse models, raising the possibility that diet could be used to redress microbiome alterations associated with disease to achieve benefits^29–33^.

In this study, we focused on dietary fiber as its consumption is much higher in developing and rural populations who have lower autoimmune disease incidence^34^. Although dietary fibers, which are composed of both fermentable and non-fermentable fibers, are essential features of a vegetarian diet^5^, earlier studies in mouse models of MS and other inflammatory diseases have mainly focused on fermentable dietary fibers. Through these studies, it has become clear that the fermentation of fibers by gut microbes increases the production of SCFA, which has well documented beneficial functions for the treatment of inflammatory disorders including EAE^7–10^.

Here we discovered that dietary non-fermentable fiber cellulose intake protected an EAE susceptible transgenic mouse strain from spontaneous autoimmune CNS disease independent of SCFAs. We found significant changes in bacterial microbiota and effector T cell responses that mediate CNS autoimmunity. Cellulose fed mice contained expanded T_H_2 cells and decreased T_H_1 cells in their immune repertoire suggesting an imbalance of immune responses. These changes are reflected in the differential recruitment of innate immune cells, eosinophils and neutrophils. Expansion of T_H_2 cells in GALT populations upon cellulose feeding was unexpected. Conventionally fed OSE mice, produce T_H_2-dependent IgG1 autoantibodies, but very little T_H_2 signature cytokines^16^. In active EAE mouse models, both T_H_1 cells along with T_H_17 cells are the main effector T cell subsets^35^ whereas the role of T_H_2 cells are unclear. While early studies found that IL-4 treated mice developed lower EAE scores along with increased T_H_2-like cells^36^, this was not seen in another EAE model^37^. In fact, T_H_2 polarized autoreactive cells transferred disease, not protection, in immunodeficient hosts^38^ and IL-4 deficient mice develop EAE indistinguishable of their wild type counterparts^39^. However, it appears that in the model used in this study, increased T_H_2 responses are partly responsible for the disease protection as neutralization of IL-4 and IL-5 only partially increased the disease incidence in cellulose rich diet fed mice. It is likely that several mechanisms act in concert for the mitigation of neurological symptoms.

Our results revealed that non-fermentable fiber supplementation in early adult life suppressed the neurological disease development. The disease suppressive effects were observed in mice that were raised and maintained in the cellulose rich diet and in mice that were switched from control diet at young age. We are not aware of any studies that has investigated the impact of dietary cellulose in EAE models. However, dietary cellulose supplementation has been shown to ameliorate DSS colitis in mice^40^. These findings support the assumption that nutritional interventions, that include non-fermentable fibers, are indeed valuable options for the autoimmune disease control.

While searching for the nutrient signals that enhances T_H_2 responses in mice fed cellulose rich diet, we have investigated microbiome and metabolic changes. Our results are consistent with previous findings that non-fermentable cellulose modify microbial composition^14,40^. We observed a slight decrease in overall bacterial diversity. In our study we found an enrichment of Ruminococcaceae, Helicobacteraceae and Enterococcaceae, and the Sutterellaceae, Lactobacillaceae and Coriobacteriaceae were reduced. Although, the low bacterial diversity has been associated with human diseases, the relative importance of diet induced microbial diversity changes and their impact on host immune system on different diseases remains to be investigated. Moreover, our findings do not support a causative role of diet-induced compositional shifts in the benefits of the CR diet in OSE mouse model. In contrast, metabolomics analysis showed an enrichment of long chain fatty acids and decrease of SCFA, butyric acid and bile acids. The decreased SCFA levels are consistent with the notion that cellulose is not fermented in the mammalian gut. However, the increase of long chain fatty acids is intriguing since they are typically present in the diet and not produced as a result gut microbial metabolism. We speculate that cellulose lowers active concentrations of bile acids by binding them, thereby affecting the rate of absorption of fatty acids in the intestine^23,24^. By testing bacterial lysates, cecal extracts, and long chain fatty acids in T_H_2 differentiation assays we confirmed that diet-induced metabolic changes rather than alterations in microbial antigens enhances T_H_2 cytokine production. Given the fact the T_H_2 cytokine neutralization did not fully restore EAE incidence *in vivo*, additional effects of the microbiome on T cells or other immune cells including antigen presenting cells that may have additive disease protective effects cannot be ruled out.

Although the precise mechanism by which long chain fatty acids enhance T_H_2 responses remains to be elucidated, this study was successful in clearly establishing cause and effect relationships. Most microbiome studies suffer from merely establishing associations between microbiome signatures and host phenotypes without consolidation of directionality^41^. By using a combination of microbiome sequencing and metabolomics with functional immune assays using cecal lysates and metabolites, we were able to identify the metabolites that are most likely responsible for the cellulose rich diet-induced benefits. Another strength of this study is the use of a spontaneous EAE model which is uniquely suited to study disease triggering processes relevant in human MS. Unlike active EAE mouse models, where resting autoreactive T cells are activated by autoantigen presentation in a hyper-inflammatory milieu created by immune adjuvant^17^, sEAE develops naturally following interaction of gut microbes and autoreactive T cells^42^.

In summary, this study provides important insights into the role of diet in a spontaneous EAE model that is relevant for human MS. Epidemiological studies have shown that countries with a high intake of saturated fat have higher risk to develop MS than in countries with a high intake of polyunsaturated fat^26^. In addition, diets rich in fiber and omega 3 fatty acids have been encouraged for MS. However, these studies are merely anecdotal, and our study clearly warrants more systematic studies in humans. Interestingly, although case-control studies have found MS risk to be associated with animal fat or animal product consumption, or a protective effect of vegetable fats, Omega-3 and 6 fatty acids showed limited effects in controlled trials^27,43^. Our finding that dietary cellulose, which is high in vegetables, is likely protective through a monounsaturated omega-9 fatty acid fatty acid (cis-11 eicosenoic acid) common in plant oil and nuts suggests a potential reason for the beneficial effects of vegetable fat. The fact that vegetarian diet can be easily consumed in daily life, makes it a useful supplementation to the currently available medications. In addition, cis-11 eicosenoic acid could be tested as a supplement or treatment in human MS. Clearly, our findings warrant more nutritional studies in human MS, a field that is severely understudied with merely anecdotal evidence despite the substantial interest among MS patients to adjust their diet to improve health prospects.

## Methods

### Mice

OSE (2D2 x IgH^MOG^) C57BL/6 and wild-type C57BL/6 mice were bred and housed in the at the animal facility of the Max Planck Institute of Biochemistry, Martinsried. Mating pairs were fed control (1314TPF or C1000) or crude fiber-rich (26% cellulose) diet (C1014) and, if not stated otherwise, offspring were maintained on the same diet as their parents. All dietary interventions were performed in the same animal rooms and experiments were matched for gender and age. Diets were γ-irradiated and purchased from Altromin, Germany. All animal procedures were in accordance with guidelines of the Committee on Animals of the Max Planck Institute of Neurobiology and with the approval from Regierung von Oberbayern (Munich, Germany).

### Cell isolation and flow cytometry

Isolation and phenotyping of immune cells by flow cytometry were done as previously described^42^. Briefly, single cell suspensions were prepared from spleens by mechanical disruption via forcing through 40 µm cell strainers (Thermo Fisher Scientific). For the isolation of lymphocytes from the small intestine, the intestine was collected in ice-cold Hank’s Balanced Salt Solution (HBSS) buffered with 15 mM Hepes. After careful removal of Peyer’s patches, fatty tissue and fecal contents, intestine was opened longitudinally and cut into small pieces. The intestinal fragments were washed three times for 15 min with stirring (350 rpm) in HBSS containing 5 mM EDTA, 15 mM Hepes and 10% fetal bovine serum (FBS). Next, intestinal pieces were washed once for 5 min with stirring in Roswell Park Memorial Institute medium (RPMI) containing 15 mM Hepes and 10% FBS, followed by an incubation step at 37 °C with stirring (550 rpm) in RPMI with 15 mM Hepes, 10% FBS and 100 U/ml Collagenase D (Roche Diagnostics). The digested tissue was washed twice in HBSS containing 5 mM EDTA, before the lymphocytes of the small intestine were resuspended in 5 ml of 40% Percoll (Sigma-Aldrich) and overlaid on 2.5 ml of 80% Percoll. Percoll gradient separation was performed by centrifugation at 780 g for 20 min at room temperature. Small intestinal lamina propria lymphocytes were harvested from the interphase of the Percoll gradient and washed once in RPMI containing 15 mM Hepes and 10% FBS.

For detection of cell surface markers, cells were stained in FACS buffer (PBS containing 1% BSA and 0.1% NaN_3_) with fluorochrome labeled Abs: APC-conjugated anti-Ly-6G (1A8), eFluor 450-conjugated anti-CD45 (30-F11), FITC-conjugated anti-Ly-6C (AL-21), PE-Cyanine 7-conjugated anti-CD11b (M1/70), PerCP-eFluor 710-conjugated anti-Siglec-F (1RNM44N) and PerCP-Cy5.5-conjugated anti-CD4 (RM4–5). For intracellular cytokine staining, cells were activated with 50 ng/ml PMA (Sigma-Aldrich) and 500 ng/ml ionomycin (Sigma-Aldrich) in the presence of 5 µg/ml brefeldin A (Sigma-Aldrich) for 4 h at 37 °C. After surface staining, cells were fixed and permeabilized using the Transcription Factor Staining Buffer Set (eBioscience) and stained intracellularly using the following antibodies: Alexa Fluor 488-conjugated anti-IL-4 (11B11), APC-conjugated anti-FoxP3 (FJK-16s), FITC-conjugated anti-IFN-γ (XMG1.2), PE-conjugated anti-IL-5 (TRFK5), PE-conjugated anti-IL-10 (JES5–16E3) and PE-conjugated anti-IL17 (TC11-18H10). All antibodies were purchased from BD Pharmingen, eBioscience or BioLegend. Cells were acquired on FACSVerse or FACSCanto (BD Biosciences) and analysis was performed using FlowJo (TreeStar) software.

### Preparation of cecal samples and 16S rRNA sequencing

DNA extraction from cecal samples was done as previously described^44^. Briefly, samples were diluted 1:10 in phosphate buffered saline (PBS) and transferred to sterile bead beating tubes (Biospec products). Cells were washed three times in ice-cold PBS. Next, 750 μl of lysis buffer (20 mM Tris [pH 8.0], 2 mM EDTA, 20 mg/ml lysozyme) were added, samples were homogenized and incubated for 20 min at 37 °C. After incubation, 85 μl of 10% sodium dodecyl sulfate solution and 40 μl proteinase K (15 mg/ml) were added and samples were incubated for 30 min at 60 °C. After addition of 500 μl of phenol-chloroform-isoamyl alcohol (25:24:1), samples were homogenized in a MiniBeadbeater-8 (BioSpec Products) for 2 min at maximum speed and placed on ice. After centrifugation, the aqueous layer was extracted twice with phenol-chloroform-isoamyl alcohol (25:24:1) and twice with chloroform-isoamyl alcohol (24:1). DNA was recovered by standard ethanol precipitation at −20 °C overnight and resuspended in 200 μl of Tris-HCl buffer (10 mM, pH 8.0).

Illumina MiSeq amplicon sequencing was done at the University of Minnesota Genomics Center. The V5-V6 region of bacterial 16S rRNA gene was amplified using the 784 F forward (5′-RGGATTAGATACCC-3′) and 1064 R reverse primer (5′-CGACRRCCATGCA NCACCT-3′)^45^. The resulting reads were trimmed to 240 bp with the FASTX-Toolkit (http://hannonlab.cshl.edu/fastx_toolkit/) and subsequently merged with the merge-illumina-pairs application (https://github.com/meren/illumina-utils) (p-value = 0.03, enforced Q30 check, perfect matching to primers and no ambiguous nucleotides were allowed). Samples exceeding 20,000 reads were subsampled to this number in Mothur v.1.31.1^46^ in order to standardize sequencing depth across samples. Sequences shorter than 240 bases or longer than 260 bases were removed. USEARCH v7.0.1001^47^ was used to generate Operational Taxonomic Units (OTUs) with a 98% similarity cut-off, and chimera sequences were removed. Samples contained an average of 18,078 ± 667 quality-controlled sequences per sample. Sequences were taxonomically characterized from phylum to genus level with Ribosomal Database Project Classifier^48^ using the MultiClassifier v1.1 tool. All phylotypes were computed as percent proportions based on the total number of sequences in each sample.

### Culture of bacteria

Frozen fecal pellets of mice were weighed, suspended and serially diluted in filter-sterilized pre-reduced phosphate buffered saline. Serial dilutions of fecal homogenates were plated on Bile-Esculin (BE) agar plates (Sigma-Aldrich). After overnight incubation at 37 °C, the colony forming units (c.f.u.) were determined and expressed as c.f.u./g of faeces.

### Bacterial DNA Extraction from mouse feces and quantitative PCR

Bacterial genomic DNA was extracted from fecal pellets using QIAamp DNA Stool mini kit (Qiagen). To validate the sequencing data, bacterial DNA was subjected to quantitative PCR using family- or strain-specific 16s rRNA primers: Enterococcaceae: forward: 5′-G TGCCAGCMGCCGCGGTAA-3′; reverse: 5′-GCCTCAAGGGCACAACCT CCAAG-3′; *E*. *faecalis*: forward: 5′-CGTGGGTAACCTACCCATCAGA-3′; reverse: 5′-AAAGC GCCTTTCACTCTTATGC-3′; *E*. *gallinarum*: forward: 5′-TCTTTCACCGGAGCTTGCTCC ATC-3′; reverse: 5′-AAAGCGCCTTTCAACTTTCTTC-3′; *E*. *hirae*: forward: 5′-TCTTTTT CCACCGGAGCTTGCTCCACCG-3′; reverse: 5′-TCAAAACCATGCGGTTTCGATTGTT AT-3′. All reactions were performed using the Absolute QPCR SYBR Green Mix (Thermo Fisher Scientific) following the manufacturer’s instructions and measured using a 7900HT Real-time PCR System (Applied Biosystems). Absolute quantity of 16S RNA gene copies was calculated using a standard curve generated with a plasmid encoding respective 16S rRNA gene. Gene expression analysis of sorted T cells from the intestine or spinal cord were performed using custom designed or pre-designed primer sets from IDT and measured using 7900HT Real-time PCR System or Quantstudio 3 PCR Real-time PCR System (Applied Biosystems).

### *In vitro* T_H_2 differentiation

Naïve CD4^+^ T cells (CD45^+^ CD4^+^ CD62L^+^ CD44^−^) were sorted and stimulated with plate-bound anti-CD3 (2 µg/ml; 145-2C11) and anti-CD28 (2 µg/ml; 37.51) in a 24-well plate for 72 h. Naïve cells were cultured at a concentration of 1 × 10^6^ cells/ml in complete RPMI-1640 with 10% FCS. For T_H_2 polarization, IL-4 (100 ng/ml) and anti-IFN-γ (10 µg/ml) were added to the cultures. To test the effect of eicosenoic acid on the T_H_2 cell response, methyl 11-cis eicosenoic acid (Sigma Aldrich) at a concentration of 25 µM or ethanol as solvent control were added to the T_H_2 cell-cultures. In some experiments, cecal lysates were added at a final concentration of 10 µg/ml.

### Preparation of cecal lysates

Cecal content of control or cellulose-rich diet-fed mice were transferred to sterile Eppendorf tubes and resuspended at 500 mg/ml in sterile PBS. Bacteria were lysed by sonication for 1 min (4 × 20 sec; amplitude 50; pulse one second). Concentrations of bacterial lysates were determined using Pierce^TM^ BCA Protein Assay Kit (Thermo Fisher Scientific).

### ELISA

Cytokines and IgE in cell culture supernatants were measured by commercially available kits from Biolegend.

### Metabolite extraction

Approximately 34 mg of wet weight of cecal content of each mouse were transferred into sterile ceramic NucleoSpin^®^ Bead Tubes (Macherey-Nagel) primed with a stainless metal bead (5 mm; Qiagen). One milliliter of ice-cold methanol was added (Sigma Aldrich), samples were homogenized in a Tissue Lyser II (Qiagen) for 5 min at 30 Hz and centrifuged for 10 min at 21,000 g and 4 °C. Supernatants were transferred into sterile Eppendorf tubes and stored at −80 °C until mass spectrometry analyses.

### Metabolomics by means of UPLC-MS analysis using (-) TOF MS/MS

LC-MS experiments were performed using an Acquity-UPLC system (Waters Milford) coupled to a SYNAPT-G1-QTOF HD mass spectrometer (Waters Micromass). A multimode column (Scherzo SM-C18; 3.0 μm, 2.0 mm × 150 mm; Imtakt) was used for chromatographic separation of polar cationic, anionic and reversed-phase suitable metabolites (multimodal RP-LC-MS). Buffers used for chromatographic separation were as followed: A: 2.5 mM ammonium acetate (Sigma-Aldrich), 0.05% acetic acid in milliQ water (pH 4.3) and B: a 1:1-mixture of acetonitrile (LC-MS CHROMASOLV, Sigma-Aldrich) and isopropanol (LC-MS CHROMASOLV, Sigma-Aldrich) with 25 mM ammoniumacetate (Sigma-Aldrich) and 0.5% acetic acid (Biosolve BV). Column oven temperature was set to 35 °C and flow rate to 250 µl/min. Injection volume was 7.5 µl. Methanolic supernatants were used for injection. Post column infusion with 2-(2-Methoxyethoxy)ethanol (2MEE; Sigma-Aldrich) to enable the detection of small polar metabolites such as butyric acid as described in detail in Koch *et al*.^49^. Post column infusion of 2-MEE was facilitated via Ultimate Plus separation system (Dionex/LC Packings, Thermo Fisher Scientific). Concentration of 2-MEE was 0.5% and 2-MEE was introduced to ESI with a flow rate of 150 μl/min. Prior to this, the flow was splited at a rate of 1:2.

Mass spectrometer had the following settings: electrospray ionization was performed in negative mode with capillary voltage of 2300 V, sample cone of 25 V, extraction cone of 4 V, source temperature of 120 °C, drying gas temperature of 350 °C, cone gas flow rate of 20 L/Hr and dessolvation gas of 800 L/Hr. Synapt mass spectrometer operated in V Mode. Scan time was set to 0.3 sec, interscan time was 0.020 sec and mass range was between 50 and 1000 Dalton. Data was acquired in centroid mode.

Data was processed in Genedata (Genedata GmbH) and submitted afterwards to MetaboAnalyst 3.0^50^. Data with features (containing mass signals and retention times) were normalized to sum of intensities, g-log transformed and unit-variance scaled. Moreover, missing values were imputed by k-Nearest neighbours and features with > 50% were removed from data analysis. These data was also imported into SIMCA to perform further analysis. Mass signals were annotated in MassTRIX (absolute error of 0.01 Dalton), a web-based server with access to three different metabolite databases: HMDB, Lipid Maps and KEGG^51^.

### Short chain fatty acids analysis

Acetic acid, propionic acid and butyric acid were purchased from Sigma Aldrich and prepared in methanol to a stock solution of 1000 ppm. SCFAs were diluted to a working concentration of 50 ppm and used for derivatization (final concentration: 5 ppm). SCFAs were derivatized using AMP + Mass Spectrometry Kit (Cayman Chemical) and subsequently analyzed by UPLC-MS in positive mode. Eight µl of each standard and cecal methanol extracts were used. After derivatization, 200 µl of methanol were added to a final concentration of 1.5 ppm. LC-MS experiments were performed using an Acquity-UPLC system (Waters Milford) coupled to Time of Flight Mass spectrometer (MaXis, Bruker Daltonics). A reversed-phase separation was applied using a C8 column (1.7 µm, 2.1 × 150 mm, Acquity^TM^ UPLC BEH^TM^, Waters, Milford). Elution of derivatized SCFAs was ensured by following solvent system of ammonium acetate (5 mM, Sigma Aldrich) combined with acetic acid (0.1%, pH 4.2, Biosolve) in water (A) and acetonitrile (B) (LC-MS CHROMASOLV®, FLUKA, Sigma Aldrich). The flowrate was 0.3 ml/min, injection volume of 1 µl and column temperature was 40 °C. A gradient profile was applied by starting at 1% of B for 1 min, increasing to 95% of B within 17 min. The 95% of B was hold for 2 min, returning to the initial 1% of B within further 0.2 min and holding 2 min with a total run time of 22 min. In addition, a pre-runtime of 2 min was included. Mass spectrometer parameters were as follows: electrospray ionization was performed in positive mode with capillary voltage of 4500 V, end plate offset of −500 V, drying gas temperature of 200 °C, dry gas of 8 l/min and nebulizer of 2.0 bar. Data was acquired in profile and centroid mode.

Peak areas were elaborated with QuantAnalysis (Bruker, Daltonics). Mass signals of SCFAs in positive mode were for acetic acid ([M]+: 227.1184), propionic acid ([M]+: 241.1341) and butyric acid ([M]+: 255.1492).

### Statistics

GraphPad Prism 6 (GraphPad Software, Inc.) was used for most of the statistical analysis. P values < 0.05 were considered to be significant. Comparisons in the abundance of bacterial taxa between the dietary treatments were performed with Wilcoxon signed-rank test and subsequent FDR correction of the p-values in R. Alpha-diversity indices (observed species, Shannon and Simpson indices) and β-diversity indices (Bray-Curtis, Morisita-Horn and binary Jaccard) were calculated in QIIME^52^. Multivariate statistical data analysis of metabolome was performed with SIMCA-P 9.0 (Umetrics). Data was scaled to unit-variance prior performing principal component analysis (PCA) or projection to latent structures by means of partial least squares discriminant analysis (PLS-DA). Mass signals responsible for calculated PLS-DA models were elaborated from VIP lists (Variable importance in the projection). Univariate statistical analysis was done in MultiExperiment Viewer 4.9 (http://mev.tm4.org), performing a Mann-Whitney U test for comparison of two groups. Student’s t-test was performed in SigmaPlot 12.0 (Systat Software GmbH). Visualization was done with Simca-P 9.0, Microsoft Office Excel or RStudio (https://www.rstudio.com/).

## Electronic supplementary material

Supplementary info
