# Supplementary material for: Dietary non-fermentable fiber prevents autoimmune neurological disease by changing gut metabolic and immune status
Source: Sci Rep. 2018 Jul 11;8:10431. doi: 10.1038/s41598-018-28839-3 (PMC6041322; doi:10.1038/s41598-018-28839-3)
Supplement: Supplementary file 1 — Supplementary info [file 41598_2018_28839_MOESM1_ESM.pdf]

Supplementary information

Dietary non-fermentable fiber prevents autoimmune neurological disease by changing gut metabolic and immune status

Kerstin Berer[1], Inés Martínez[2], Alesia Walker[3], Birgit Kunkel[1,7], Philippe Schmitt-Kopplin[3,4,5], Jens Walter[2,6] and Gurumoorthy Krishnamoorthy[7]*

[1]Hertie Senior Professor Group, Max Planck Institute of Neurobiology, Martinsried, Germany

[2]Department of Agricultural, Food & Nutritional Science, University of Alberta, Edmonton, Canada

[3]Research Unit Analytical BioGeoChemistry, Helmholtz Zentrum München, Neuherberg, Germany

[4]Chair of Analytical Food Chemistry, Technische Universität München, Freising-Weihenstephan, Germany

[5]ZIEL – Institute for Food & Health, Technische Universität München, Germany

[6]Department of Biological Sciences, University of Alberta, Edmonton, Canada

[7]Research group Neuroinflammation and mucosal Immunology, Max Planck Institute of Biochemistry, Martinsried, Germany

*Corresponding author. E-mail: guru@biochem.mpg.de

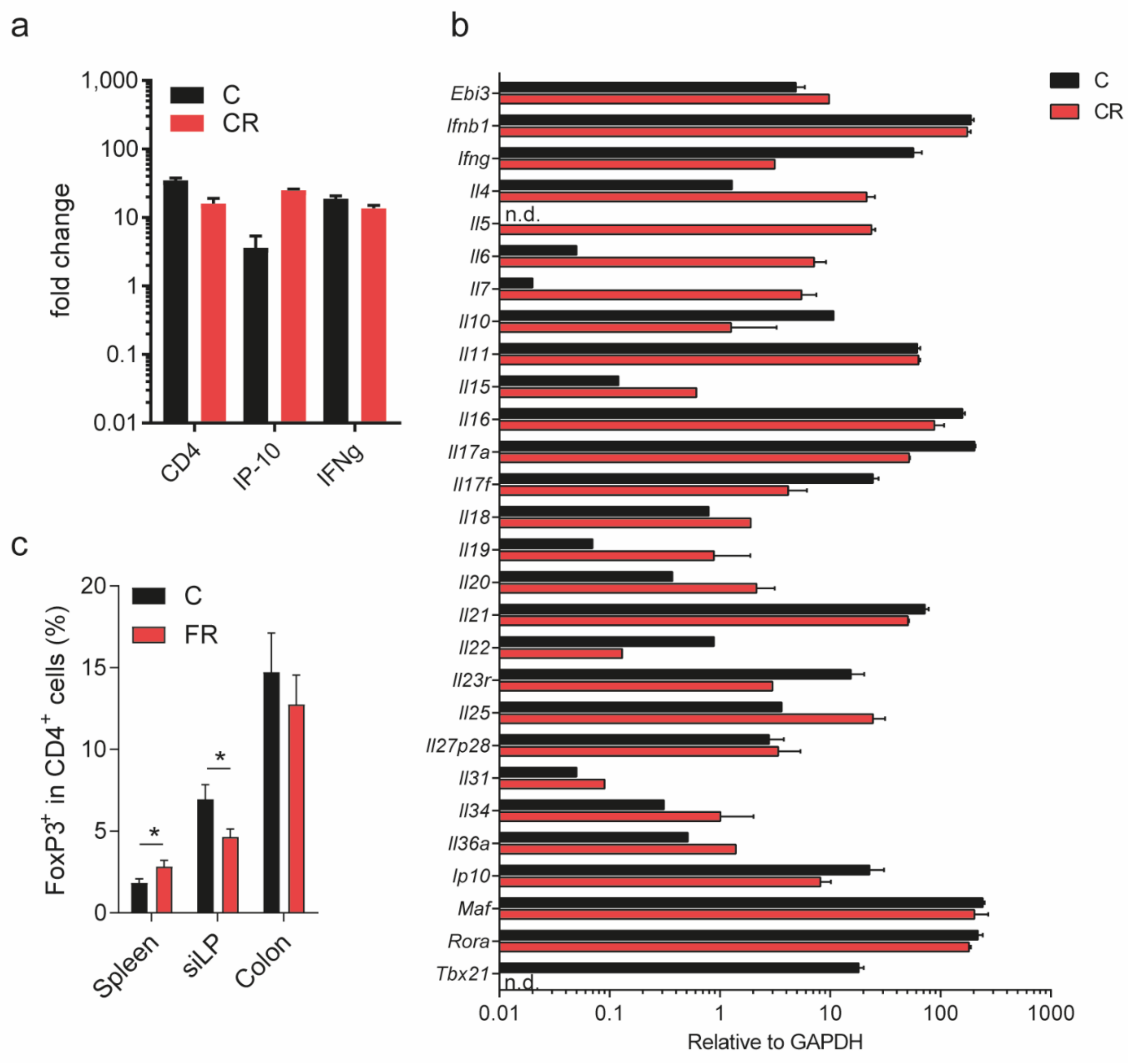

Supplementary Figure 1 Immune signature in T cells from mice fed a control or cellulose rich diet. (**a**) Analysis of gene expression in the spinal cord of OSE mice fed control (C) or cellulose-rich diet (CR). Expression levels of CD4, IP-10, and IFNγ genes were quantified by real-time PCR. Fold changes were calculated relative to healthy mice spinal cords. *n* = 2-4 mice per group. (**b**) Gene expression profile of $CD4^+$ T cells. $CD4^+$ T cells were isolated from small intestinal lamina propria and expression levels of various cytokines and transcription factors were quantified by real-time PCR. n.d. = not detectable. Data were pooled from 3 independent experiments. (**c**) Frequencies of $FoxP3^+$ T_{reg} cells from the indicated organs of mice fed control (C) or cellulose-rich diet (CR) are displayed. siLP, small intestinal lamina propria. *n* = 11-12 mice per group. Data were pooled from 3 independent experiments. **P* < 0.05 (Mann-Whitney U test).

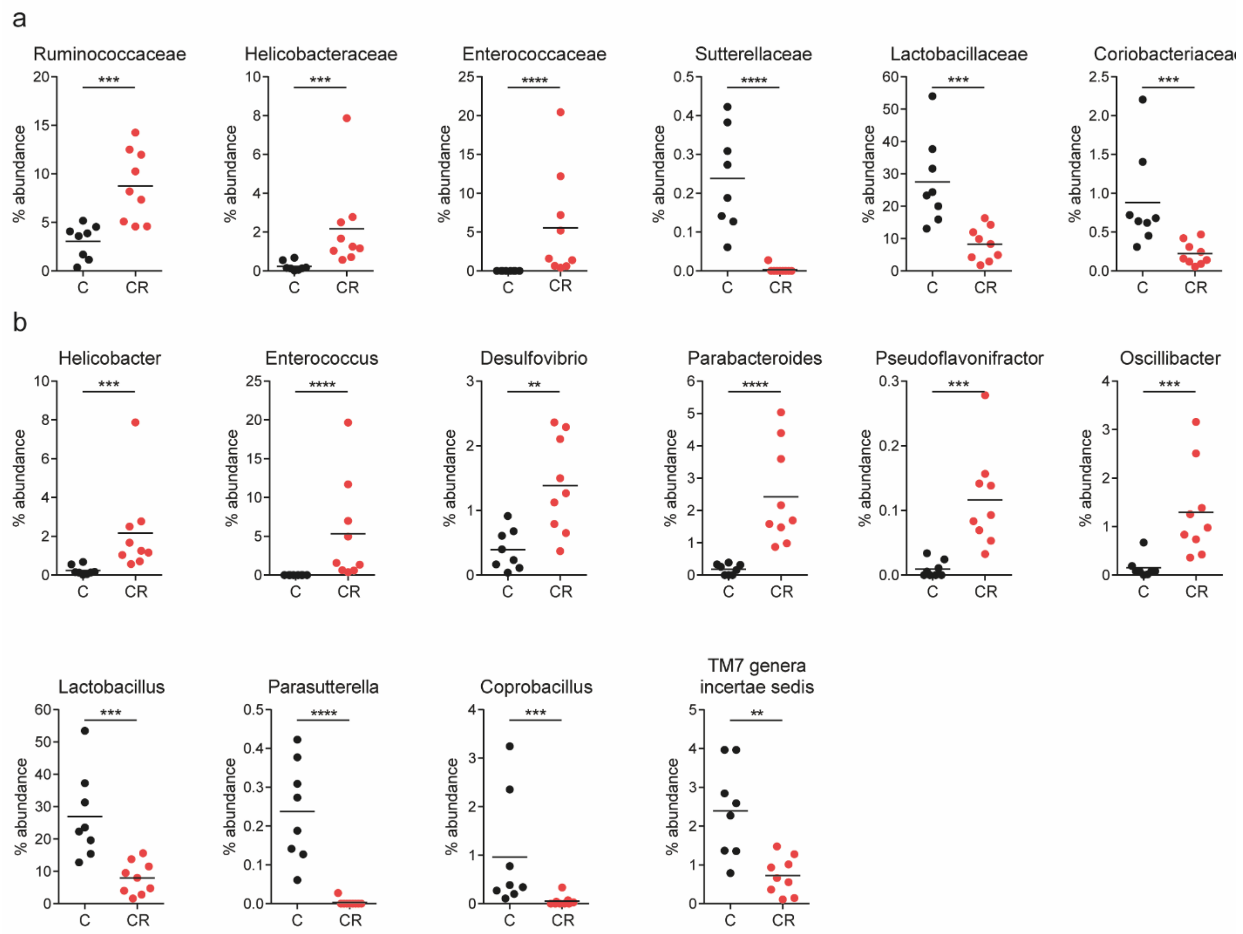

Supplementary Figure 2 Dietary cellulose alters the composition of the intestinal microbiota. (**a**) Relative abundances of bacterial families Ruminococcaceae, Helicobacteraceae, Enterococcaceae, Sutterellaceae, Lactobacillaceae and Coriobacteriaceae in the feces of control (C; *n* = 8) or CR diet-fed mice (CR; *n* = 9). ***$P < 0.001$; ****$P < 0.0001$ (Mann-Whitney U test). (**b**) Relative abundances of the bacterial genera *Helicobacter*, *Enterococcus, Desulfovibrio, Parabacteroides, Pseudoflavonifractor*, *Oscillibacter*, *Lactobacillus, Parasutterella, Coprobacillus* and *TM7 genera Incertae Sedis* in the feces of control (C; *n* = 8) or CR diet-fed mice (*n* = 9). **$P < 0.01$; ***$P < 0.001$; ****$P < 0.0001$ (Mann-Whitney U test).

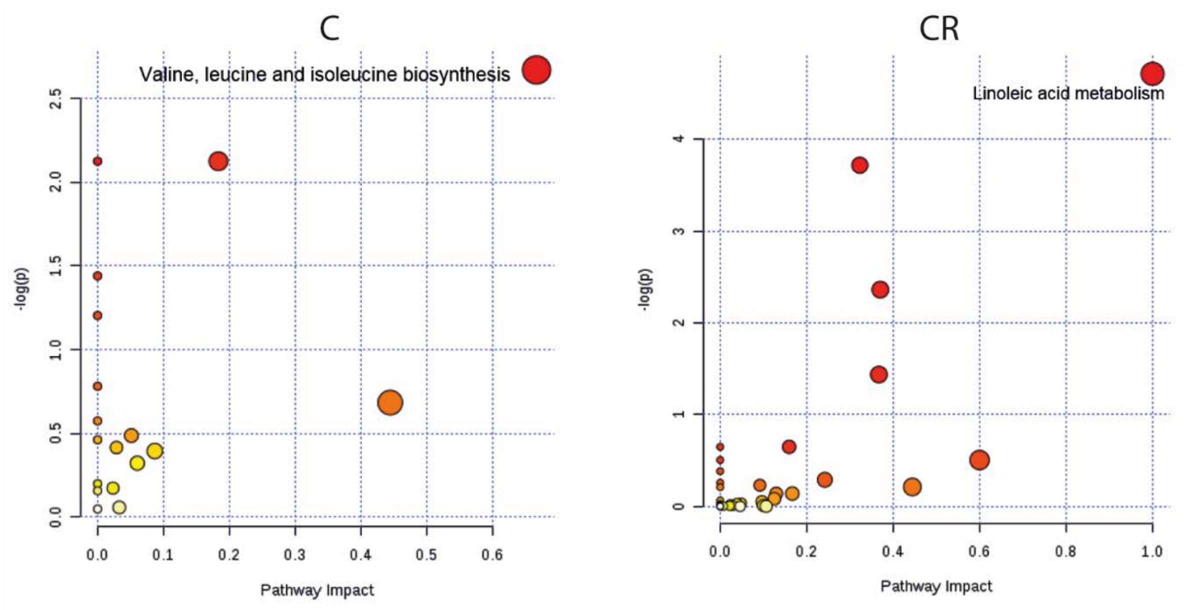

Supplementary Figure 3 Dietary cellulose alters the cecal metabolites profile. (**a**) KEGG pathways enrichment analysis were performed on metabolites of the ceca of mice raised on control (C; *n* = 8) or CR diet (*n* = 10). (**b**) SCFAs, i.e. acetic, propionic and butyric acids, are significantly changed between control (C; *n* = *3*) and CR diet-fed mice (*n* = 4); ****P* < 0.001 (Student's t-test).

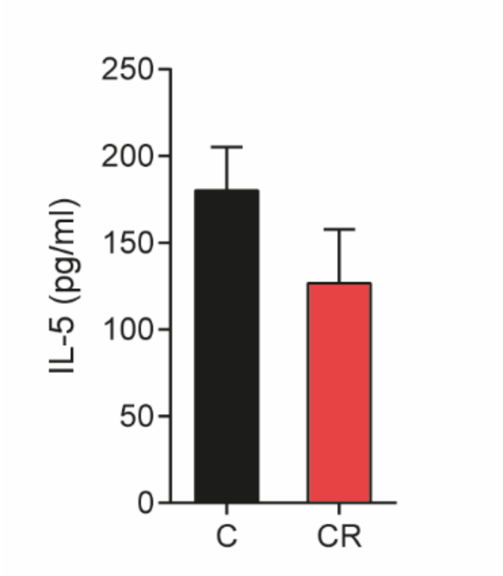

Supplementary Figure 4 Cecal bacterial lysates does not affect T_H2 immune response. Naïve $CD4^+$ T cells were stimulated under T_H2 cell polarizing conditions in the presence of cecal lysates (10µg/ml) of control (C lysates) or CR (CR lysates) diet-fed mice. Levels of IL-5 in the supernatants were measured by ELISA. Bars display mean ± s.e.m. $n = 3$ independent experiments.

Supplementary Table 1. Abundance profiles of bacterial taxa in control (C) and cellulose-rich (CR) diet-fed mice.

	Control (mean ± sd)	CR (mean ± sd)	P-value (FDR corrected)
**Phylum**			
Proteobacteria	2.68 ± 2.05	9.11 ± 4.17	0.025
TM7	2.39 ± 1.19	0.73 ± 0.48	0.028
**Family**			
Ruminococcaceae	3.06 ± 1.75	8.76 ± 3.67	0.014
Lactobacillaceae	27.51 ± 13.37	8.29 ± 5.19	0.014
Enterococcaceae	0.00 ± 0.00	5.52 ± 6.87	0.014
Helicobacteraceae	0.24 ± 0.24	2.17 ± 2.27	0.014
Coriobacteriaceae	0.88 ± 0.63	0.22 ± 0.15	0.027
Desulfovibrionaceae	0.57 ± 0.37	2.15 ± 1.08	0.028
Sutterellaceae	0.24 ± 0.13	0.00 ± 0.01	0.014
**Genus**			
Lactobacillus	26.98 ± 13.36	7.97 ± 5.00	0.014
Enterococcus	0.00 ± 0.00	5.32 ± 6.59	0.014
Parabacteroides	0.18 ± 0.16	2.42 ± 1.53	0.014
Helicobacter	0.24 ± 0.24	2.17 ± 2.27	0.014
Oscillibacter	0.15 ± 0.22	1.29 ± 0.95	0.023
Pseudoflavonifractor	0.01 ± 0.01	0.12 ± 0.07	0.014
Coprobacillus	0.96 ± 1.18	0.05 ± 0.11	0.025
TM7 genera Incertae Sedis	2.39 ± 1.19	0.73 ± 0.48	0.028
Desulfovibrio	0.39 ± 0.31	1.38 ± 0.73	0.040
Parasutterella	0.24 ± 0.13	0.00 ± 0.01	0.014
**OTUs**<br>**Taxonomic assignment***			
OTU_101<br>Burkholderiaceae, HM124111.1 (100%)	0.25 ± 0.14	0.00 ± 0.00	0.020
OTU_11<br>Lachnospiraceae, JQ084455.1 (99%)	0.01 ± 0.02	4.08 ± 3.39	0.021
OTU_114<br>Firmicutes, AB702888.1 (100%)	0.22 ± 0.12	0.00 ± 0.00	0.020
OTU_115<br>Lachnospiraceae, JQ085234.1 (100%)	0.22 ± 0.22	0.01 ± 0.02	0.021
OTU_117<br>Ruminococcaceae, JQ085043.1 (100%)	0.22 ± 0.27	0.00 ± 0.00	0.041
OTU_118<br>Clostridium IV, EU457476.1 (100%)	0.00 ± 0.00	0.17 ± 0.22	0.041

OTU_12<br>*Helicobacter* sp., DQ019255.1 (100%)<br>*Helicobacter apodemus*, AY009129.1 (98%)<br>*Helicobacter canadensis*, KJ534296.1 (98%)	0.13 ± 0.15	1.89 ± 2.11	0.021
OTU_12<br>Porphyromonadaceae, FJ674457.1 (95%)	0.18 ± 0.20	0.00 ± 0.00	0.020
OTU_124<br>*Clostridium* XIVa, EU453012.1 (99%)	0.00 ± 0.00	0.18 ± 0.12	0.021
OTU_13<br>Lachnospiraceae, JQ084127.1 (100%)	0.05 ± 0.08	1.85 ± 1.23	0.021
OTU_14<br>Lachnospiraceae, HM811058.1 (100%)	4.66 ± 5.14	0.01 ± 0.02	0.021
OTU_142<br>Coriobacteriaceae, EU456594.1 (100%)<br>*Denitrobacterium* sp., AJ518870.1 (98%)<br>*Gordonibacter* sp., KF785806.1 (98%)	0.13 ± 0.27	0.00 ± 0.00	0.041
OTU_144<br>Clostridiales Incertae Sedis XIII, AB702885.1 (100%)	0.15 ± 0.16	0.00 ± 0.00	0.041
OTU_15<br>*Alistipes*, EU656100.1 (100%)	2.07 ± 1.82	0.00 ± 0.00	0.021
OTU_155<br>Coriobacteriaceae, EU457418.1 (99%)	0.18 ± 0.15	0.00 ± 0.00	0.021
OTU_158<br>Lachnospiraceae, EU455294.1 (100%)	0.15 ± 0.16	0.00 ± 0.00	0.041
OTU_165<br>Porphyromonadaceae, FJ674457.1 (95%)	0.10 ± 0.08	0.00 ± 0.00	0.041
OTU_17<br>*Desulfovibrio* sp., AF056090.1 (100%)<br>*Desulfovibrio fairfleldensis*, U42221.1 (99%)	0.39 ± 0.31	1.38 ± 0.73	0.048
OTU_178<br>Lachnospiraceae, EU454918.1 (100%)	0.00 ± 0.00	0.09 ± 0.10	0.041
OTU_18<br>Parabacteroides, AB702753.1 (100%)	0.12 ± 0.11	1.17 ± 0.96	0.027
OTU_19<br>TM7 genera Incertae Sedis, FJ879268.1 (100%)	2.15 ± 1.10	0.66 ± 0.46	0.041
OTU_190<br>*Barnesiella*, EF614530.1 (99%)	0.15 ± 0.12	0.00 ± 0.00	0.021
OTU_195<br>Porphyromonadaceae, EU511738.1 (100%)	0.09 ± 0.11	0.00 ± 0.00	0.021
OTU_20<br>Ruminococcaceae, AB702871.1 (100%)	0.11 ± 0.13	1.56 ± 1.05	0.021
OTU_21<br>Ruminococcaceae, AB702876.1 (100%)	0.11 ± 0.18	1.14 ± 0.89	0.027
OTU_210<br>Coriobacteriaceae, EF614565.1 (99%)<br>*Adlercreutzia equolifaciens*, NR_121696.1 (97%)<br>*Assacharobacter celatus*, NR_114402.1 (97%)	0.09 ± 0.18	0.00 ± 0.00	0.041
OTU_22<br>Lachnospiraceae, HM817816.1 (100%)	0.02 ± 0.04	1.70 ± 0.80	0.021

OTU_23<br>*Alistipes*, JQ085082.1 (100%)	0.08 ± 0.13	1.00 ± 0.45	0.021
OTU_25<br>*Bacteroides stercorirosoris*, AB574479.1 (100%)<br>*Bacteroides oleiciplenus*, NR_112895.1 (100%)<br>*Bacteroides intestinalis*, NR_041307.1 (98%)	0.01 ± 0.03	0.95 ± 1.72	0.034
OTU_258<br>Lachnospiraceae, JQ085228.1 (100%)	0.12 ± 0.17	0.00 ± 0.00	0.041
OTU_26<br>Lachnospiraceae, HM821951.1 (100%)	0.00 ± 0.00	1.19 ± 1.29	0.024
OTU_263<br>*Parvibacter caecicola*, NR_117374.1 (100%)<br>*Enterorhabdus mucosicola*, KC206034.1 (98%)	0.05 ± 0.04	0.00 ± 0.00	0.041
OTU_264<br>Lachnospiraceae, JQ084976.1 (100%)	0.00 ± 0.01	0.15 ± 0.12	0.041
OTU_268<br>Lachnospiraceae, AB627655.1 (100%)	0.06 ± 0.07	0.00 ± 0.00	0.041
OTU_28<br>Uncultured bacterium, JQ084933.1 (100%)<br>*Coprobacillus cateniformis*, GU811876.1 (98%)	0.98 ± 1.23	0.00 ± 0.00	0.020
OTU_280<br>*Prevotella*, JQ084868.1 (100%)<br>*Prevotella brevis*, AB501176.1 (97%)	0.05 ± 0.05	0.00 ± 0.00	0.020
OTU_294<br>Coriobacteriaceae, DQ015493.1 (100%)<br>*Gordonibacter* sp., KF785806.1 (98%)	0.03 ± 0.04	0.00 ± 0.01	0.041
OTU_3<br>Lachnospiraceae, AB627586.1 (100%)	0.00 ± 0.00	7.53 ± 6.45	0.041
OTU_30<br>*Alistipes*, AB702771.1 (100%)	1.85 ± 1.84	0.02 ± 0.07	0.021
OTU_34<br>*Desulfovibrio*, JQ084347.1 (100%)	0.75 ± 1.35	0.00 ± 0.00	0.020
OTU_37<br>*Parabacteroides goldstenii*, NR_113076.1 (100%)	0.00 ± 0.01	0.61 ± 0.71	0.039
OTU_39<br>Lachnospiraceae, EU656088.1 (100%)	0.90 ± 0.62	0.00 ± 0.00	0.020
OTU_45<br>Lachnospiraceae, EF098480.1 (100%)	0.00 ± 0.00	0.58 ± 0.37	0.021
OTU_454<br>*Barnesiella*, AB702761.1 (99%)	0.02 ± 0.02	0.00 ± 0.00	0.041
OTU_5<br>*Enterococcus durans*, KJ702539.1 (100%)<br>*Enterococcus faecium*, KJ702553.1 (100%)<br>*Enterococcus mundtii*, KJ607189.1 (100%)<br>*Enterococcus avium*, AB932546.1 (100%)<br>*Enterococcus hirae*, KJ081452.1 (100%)	0.00 ± 0.00	5.47 ± 6.77	0.021
OTU_51<br>Ruminococcaceae, HM835697.1 (100%)	0.01 ± 0.03	0.49 ± 0.46	0.021
OTU_523<br>Lachnospiraceae, HM817817.1 (99%)	0.12 ± 0.17	0.00 ± 0.00	0.041

OTU_56<br>Uncultured bacterium, AB606384.1 (100%)<br>*Eubacterium infirmum*, HM596276.1 (97%)	0.51 ± 0.37	0.00 ± 0.00	0.020
OTU_560<br>Lachnospiraceae, EU656066.1 (99%)	0.06 ± 0.04	0.00 ± 0.00	0.021
OTU_6<br>*Desulfovibrio*, AB606308.1 (100%)	0.51 ± 0.54	4.41 ± 2.85	0.022
OTU_64<br>*Clostridium* XIVa, EU453765.1 (100%)	0.02 ± 0.03	0.32 ± 0.17	0.032
OTU_66<br>Porphyromonadaceae, HQ740441.1 (100%)	0.44 ± 0.36	0.00 ± 0.00	0.020
OTU_67<br>Lachnospiraceae, AB702807.1 (100%)	1.20 ± 1.58	0.00 ± 0.00	0.041
OTU_700<br>Ruminococcaceae, EF097820.1 (100%)	0.02 ± 0.02	0.00 ± 0.00	0.021
OTU_71<br>Lachnospiraceae, HQ746680.1 (100%)	0.78 ± 0.74	0.03 ± 0.08	0.025
OTU_719<br>Lachnospiraceae, JQ083958.1 (100%)	0.00 ± 0.01	0.05 ± 0.05	0.041
OTU_74<br>Lachnospiraceae, AB626912.1 (100%)	0.40 ± 0.52	0.00 ± 0.00	0.041
OTU_742<br>TM7 genera Incertae Sedis, HQ701044.1 (100%)	0.03 ± 0.02	0.00 ± 0.01	0.021
OTU_77<br>Lachnospiraceae, HM817078.1 (100%)	0.00 ± 0.01	0.31 ± 0.23	0.021
OTU_8<br>*Lactobacillus intestinalis*, JF923643.1 (100%)<br>*Lactobacillus helveticus*, KF030750.1 (99%)<br>*Lactobacillus acidophilus*, KF548315.1 (99%)	3.75 ± 4.67	0.00 ± 0.00	0.021
OTU_811<br>Erysipelotrichaceae, EU510291.1 (99%)	0.27 ± 0.30	0.00 ± 0.00	0.041
OTU_84<br>Rikenellaceae, AB702772.1 (100%)	0.36 ± 0.52	0.00 ± 0.01	0.041
OTU_86<br>Lachnospiraceae, HM818016.1 (100%)	0.00 ± 0.00	0.39 ± 0.35	0.021
OTU_88<br>Lachnospiraceae, AB702804.1 (100%)	0.34 ± 0.30	0.00 ± 0.00	0.020
OTU_894<br>Lachnospiraceae, JQ084418.1 (99%)	1.27 ± 1.85	0.01 ± 0.01	0.021
OTU_900<br>*Alistipes*, HQ740394.1 (99%)	0.09 ± 0.12	0.00 ± 0.00	0.021
OTU_903<br>*Streptococcus gallolyticus*, KJ160184.1 (99%)	0.02 ± 0.01	0.07 ± 0.05	0.041
OTU_911<br>*Streptococcus macedonicus*, KF147886.1 (99%)	0.01 ± 0.03	0.94 ± 0.72	0.021
OTU_92<br>*Streptococcus pasteuranius*, JN581989.1 (99%)	0.42 ± 0.41	0.00 ± 0.00	0.032
OTU_95<br>*Clostridium* XIVa, JQ083971.1 (100%)	0.01 ± 0.03	0.36 ± 0.33	0.021

OTU_99<br>Lachnospiraceae, HM813376.1 (100%)	0.01 ± 0.01	0.23 ± 0.24	0.041

*Taxonomic assignment of a representative sequence of the OTU at the deepest taxonomic rank possible (≥80% bootstrap cutoff in RDP Classifier, or ≥98% homology to NCBI classified sequence).
